# Supplementary material for: Crossover from gas-like to liquid-like molecular diffusion in a simple supercritical fluid
Source: Nat Commun. 2024 May 16;15:4142. doi: 10.1038/s41467-024-47961-7 (PMC11099187; doi:10.1038/s41467-024-47961-7)
Supplement: Supplementary file 1 — Supplementary Information [file 41467_2024_47961_MOESM1_ESM.pdf]

Supplementary Information

Crossover from gas-like to liquid-like molecular diffusion in a simple supercritical fluid

Ranieri, U. et al.

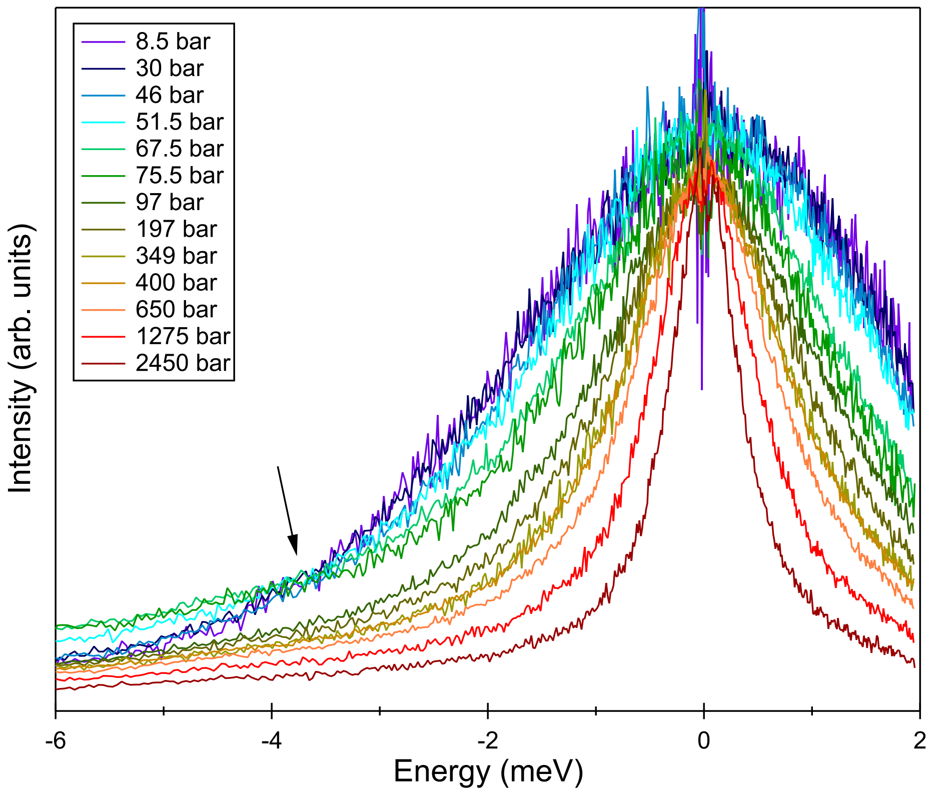

Supplementary Figure 1: Examples of QENS spectra. QENS spectra of the sample at Q=1 Å^{-1} and the indicated pressures. Spectra were arbitrarily normalized for having the same height at zero energy transfer. Note that the pressure-induced Gaussian-to-Lorentzian change in the quasi-elastic lineshape leads to the visible crossing of the spectra at about -3.5 meV, which is also indicated by the arrow.

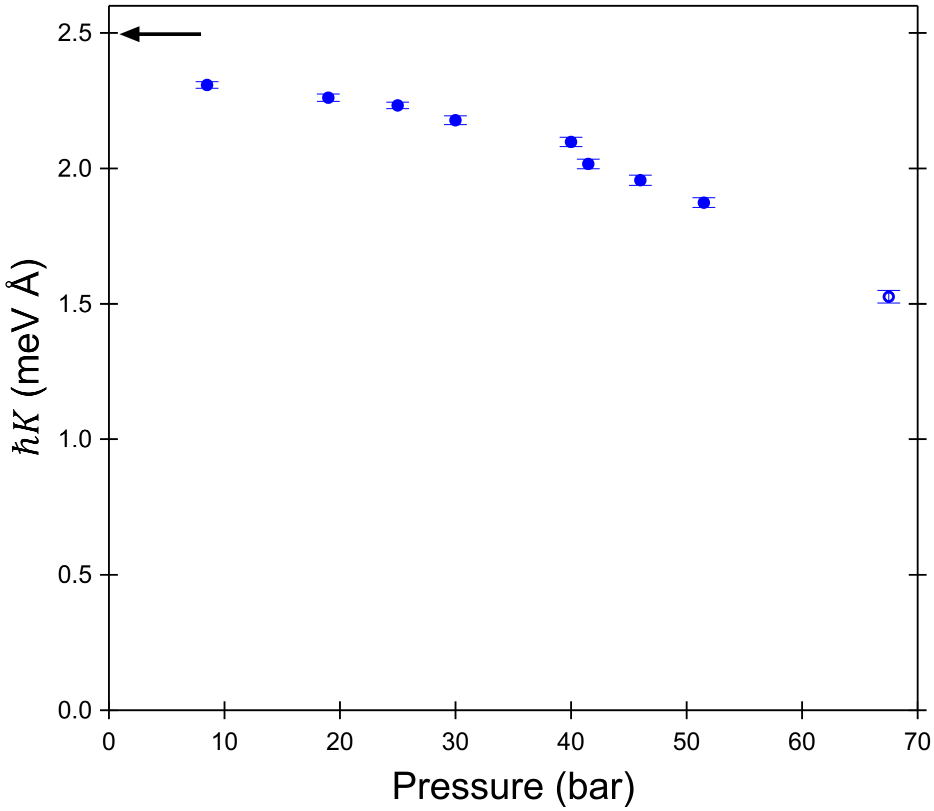

Supplementary Figure 2: Slope parameter K appearing in Eq. 4 of the main text. Pressure dependence of the slope of the linear fits of the Gaussian half widths shown in Fig. 3a of the main text. 67.5 bar is also included. The arrow indicates the value calculated from the theoretical expression for ballistic diffusion (Eq. 1 of the main text): $K = (2\ln(2)k_{\mathrm{B}}T/m)^{1/2}$.

Supplementary Table 1. Pressure P (measured within a 1 bar uncertainty) and density ρ deduced from the literature equation of state as well as slope K, self-diffusion coefficient D, and residence time τ with their fractional uncertainties (one standard deviation) as well as literature shear viscosity η. All columns are for methane at 200 K.

P (bar)	ρ (g cm^{-3})	$\hbar K$ (meV Å)	$\delta(\hbar K)(\%)$	D (10^{-9} m^2 s^{-1})	$\delta D(\%)$	τ (ps)	$\delta\tau(\%)$	η (10^{-6} Pa s)
8.5	0.00868	2.31	0.52	1481	0.52			7.98
19	0.02109	2.26	0.60	580	0.60			8.29
25	0.02935	2.23	0.54	404	0.54			8.53
30	0.03715	2.18	0.75	306	0.75			8.78
40	0.05672	2.10	0.82	184	0.82			9.47
41.5	0.06036	2.02	0.88	165	0.88			9.61
46	0.07303	1.96	0.98	128	0.98			10.14
51.5	0.09470	1.87	0.95	89.4	0.95			11.18
67.5	0.21249			36.0	0.30	0.173	0.80	21.00
75.5	0.23455			33.5	0.42	0.181	1.18	24.04
97	0.26347			29.05	0.26	0.155	0.95	28.88
197	0.31301			22.25	0.29	0.147	1.37	40.23
349	0.34616			17.83	0.32	0.134	1.97	50.75
400	0.35399			17.50	0.19	0.138	1.16	53.67
650	0.38243			14.37	0.21	0.136	1.51	65.99
1275	0.42482			10.24	0.27	0.139	2.45	90.56
2450	0.47101			6.80	0.30	0.149	3.66	130.00

Supplementary Note 1: Locating the Gaussian-to-Lorentzian crossover upon pressure increase

The Gaussian-to-Lorentzian crossover cannot be easily located in pressure based on the comparison of the chi-square values of the fits of the spectra alone, which are Q dependent and also partially dependent on other fitting parameters. Some other considerations can be made to locate the crossover, as reported here below, and the chi-square will be discussed in the context of the Bayesian analysis of the spectra (Supplementary Note 2).

First, we observe that for a given wavevector transfer value, the normalized experimental spectra cross as a consequence of the pressure-induced Gaussian-to-Lorentzian change in the quasi-elastic lineshape. This is shown for example for Q=1 Å^{-1} in Supplementary Fig. 1, where the crossing happens at about -3.5 meV when comparing the 46 bar and 51.5 bar spectra as well as the 51.5 bar and the 67.5 bar spectra. This would then locate the Gaussian-to-Lorentzian crossover in between 46 and 67.5 bar.

Second, even though the Gaussian and Lorentzian fits to the spectra are of comparable quality at 40–60 bar (see Fig. 2 of the main text), for the Lorentzian fits, the flat background had to be imposed to zero (it would otherwise converge to negative values), as shown in Supplementary Fig. 13.

Third, being Q-independent, the likeliness of the diffusion coefficients obtained from the

Gaussian and Lorentzian fits is a rather strong indication of the crossover. As briefly mentioned in the main text in relation to Fig. 4, the largest relative deviations between our experimental D and the literature[1] molecular dynamics hard-spheres prediction for D are for the intermediate pressure range (40-80 bar). This is quantified in Supplementary Fig. 3, which indeed shows the relative deviations as a function of pressure and includes the Lorentzian estimations at 46 and 51.5 bar, and the Gaussian estimation at 67.5 bar. Given that the hard spheres results are very precise and well established, this approach provides a robust indication in locating the crossover at about 60 bar (see Supplementary Fig. 3).

Finally, on a purely theoretical ground, particles in a system are expected to behave as free when probed over a spatial range much shorter than the mean free path l_0=$\left(\pi\sqrt{2}n\sigma^2\right)^{-1}$. While this should be only taken as a rough indication, we notice that 51.5 bar is our highest investigated pressure for which we employed the Gaussian fits and also the highest investigated pressure for which l_0Q >1 over the entire Q range of the present experiment, i.e. 0.4–1.5 $Å^{-1}$ (see plots in Supplementary Fig. 4). At 65 bar, l_0Q is slightly smaller than unity for our smallest Q value. At our highest pressure (2450 bar), l_0Q is smaller than 1 over most of the investigated Q range.

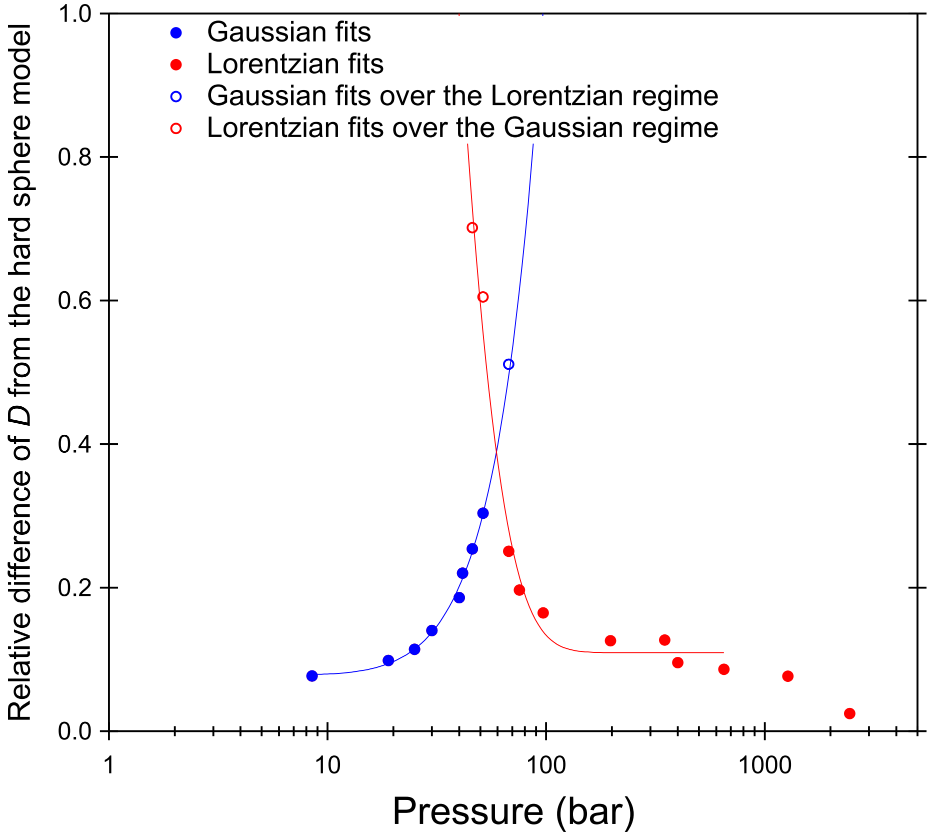

Supplementary Figure 3: Relative deviation of D from the hard sphere prediction. Relative difference of our experimental diffusion coefficient D from the hard sphere prediction at the same pressure (both reported in Fig. 4 of the main text), plotted as a function of pressure. From 46 to 67.5 bar, results from both models are reported. The two lines (guides to the eye) cross at about 60 bar.

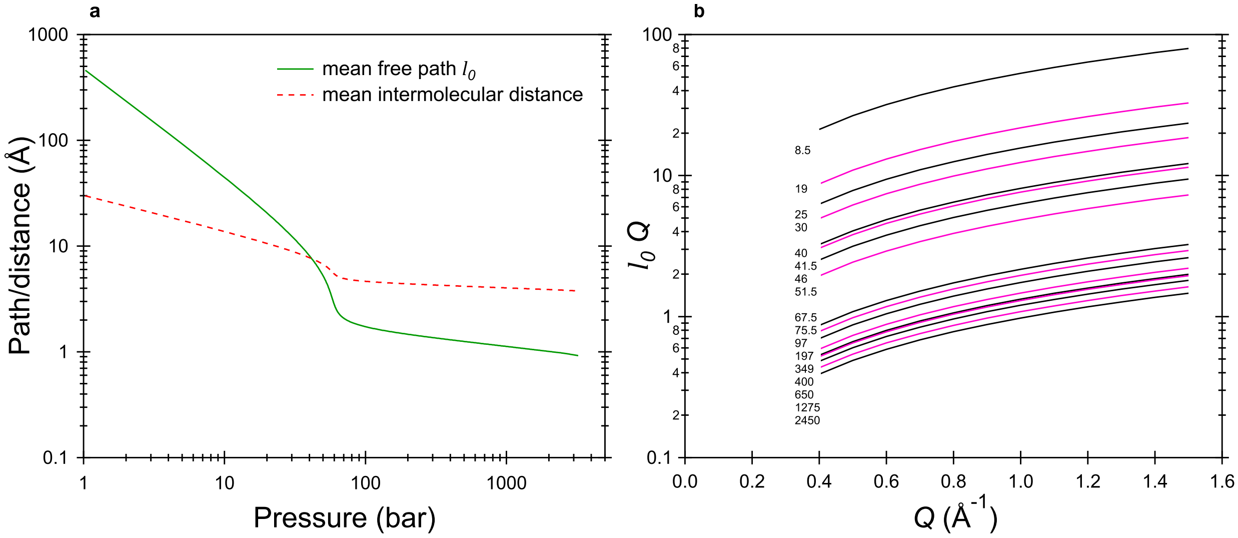

Supplementary Figure 4: Mean free path. Panel **a**: Mean free path l_0 at 200 K as a function of pressure, calculated using a molecular diameter of 3.61 Å. The mean intermolecular distance (given by $n^{-1/3}$ with n the number density) is also reported and the two lines cross at about 42 bar. Panel **b**: Product l_0Q as a function of Q at each investigated pressure of this study.

Supplementary Note 2: Bayesian analysis of the spectra

To further validate the results of our analysis, we re-analysed a data subset using a Bayesian approach, adapting a homemade code for the analysis of neutron and x-ray Brillouin scattering spectra[2]. This approach allowed us to verify two important aspects of our analysis. Firstly, it enabled us to establish the number of diffusive modes identifiable from our QENS measurements. Secondly, it provided a second independent description of the spectral lineshapes to confirm whether they exhibit a more Gaussian or Lorentzian-like nature as the pressure in the system varies. The use of Markov chain Monte Carlo methods[3] augmented by a Reversible Jump routine[4] allows us to determine the posterior distribution function of the number of spectral lines contributing to the measured scattering signal. This enables us to quantitatively and probabilistically define the number of detectable diffusive modes conditionally on the collected experimental data in an unbiased manner.

For all the spectra analysed, by employing a uniform prior for the number of potential contributions in the spectrum to adopt the least invasive approach in determining the best fitting function, the result is unequivocal, as the posterior probability $P(k = 1|y)$ of observing a single diffusive mode in the spectrum conditionally on the observed data is always greater than 95%. The same fitting algorithm naturally allows for the estimation of the parameters of the model most visited by the algorithm as well as for the background, primarily using the Metropolis-Hastings method for parameter updates[5]. For each parameter of the model, there will be a posterior distribution func-

tion from which either the mean value or the statistical mode can be considered, as appropriate, to determine the description of the lineshape most probabilistically justified by the data. For a direct comparison with the presented work, after fitting each dataset with the Bayesian algorithm either with a Gaussian profile or with a Lorenztian one, albeit unorthodox in the context of Bayesian inference, we determined also the chi-square for each of the fits performed. We could thus obtain an independent evidence about the progressive change from a Gaussian-like to a Lorentzian-like nature for the QENS spectra of methane as pressure increases.

The main outcomes of this analysis are:

1. At low pressures (P<40 bar), the Gaussian model is always preferable to the Lorentzian one for Q values lower than 1.1 Å^{-1}, while they are equivalent for higher Q values. An example for the lowest pressure value (8.5 bar) is reported below in Supplementary Figs. 5 and 6.

2. In the intermediate P range 40–60 bar, the Gaussian model is preferable to the Lorentzian one for Q values lower than 1 Å^{-1} (i.e. the region where the diffusion coefficient is evaluated), while there is a region between 1 and 1.3 Å^{-1} where Lorentzian seems preferable, and above they are comparable. As an example, the results of the Bayesian analysis for 46 bar are reported below in Supplementary Figs. 7 and 8.

3. At high pressures (P>67 bar), the Lorentzian fit is preferable for each measured Q value. As an example, the results of the Bayesian analysis for 67.5 bar are reported below in Supplementary Figs. 9 and 10.

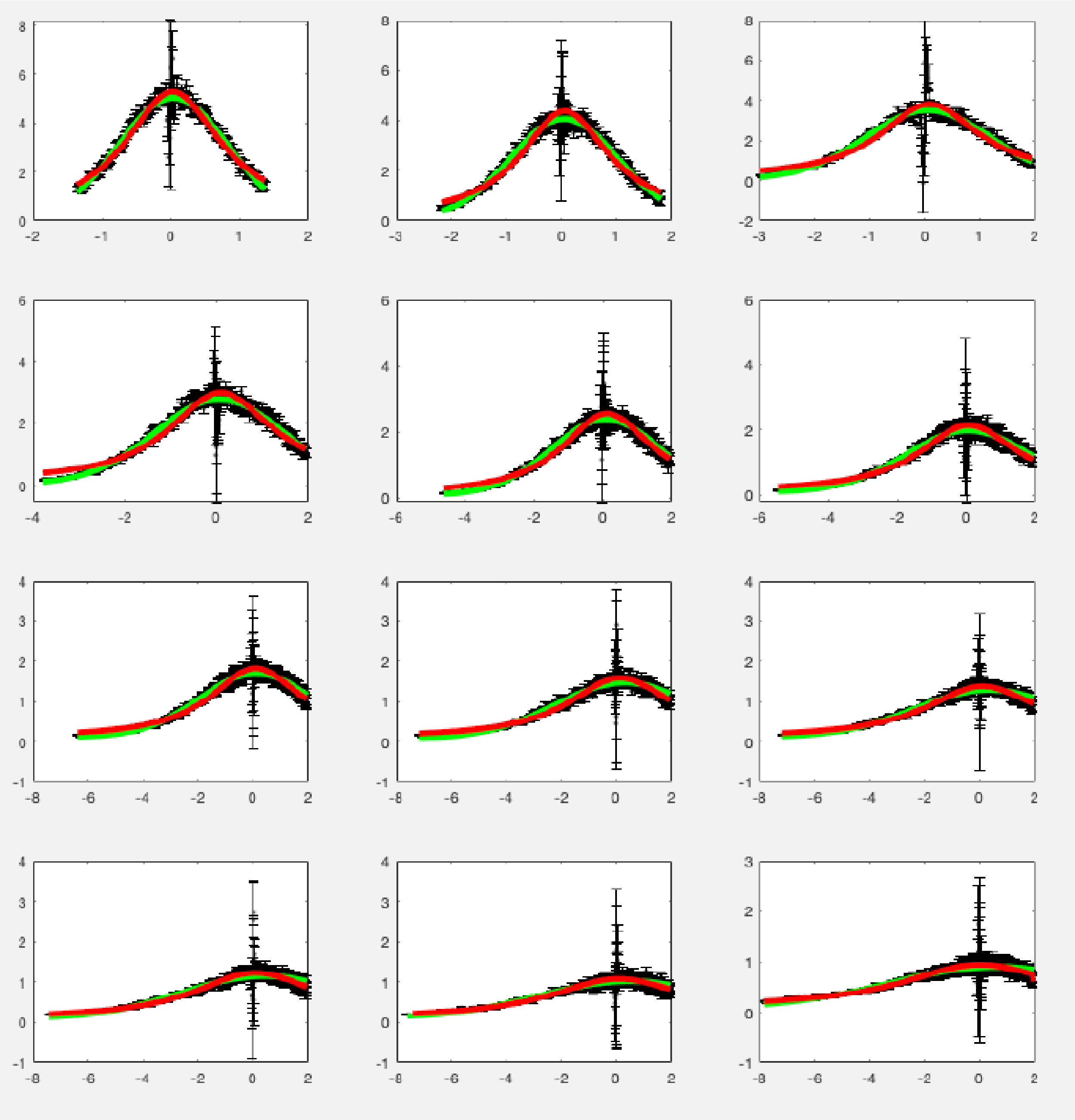

Supplementary Figure 5: Bayesian fits to the spectra at 8.5 bar. Gaussian (green) and Lorentzian (red) fits to the experimental spectra at 0.4 to 1.5 Å^{-1} (from left to right, top to bottom).

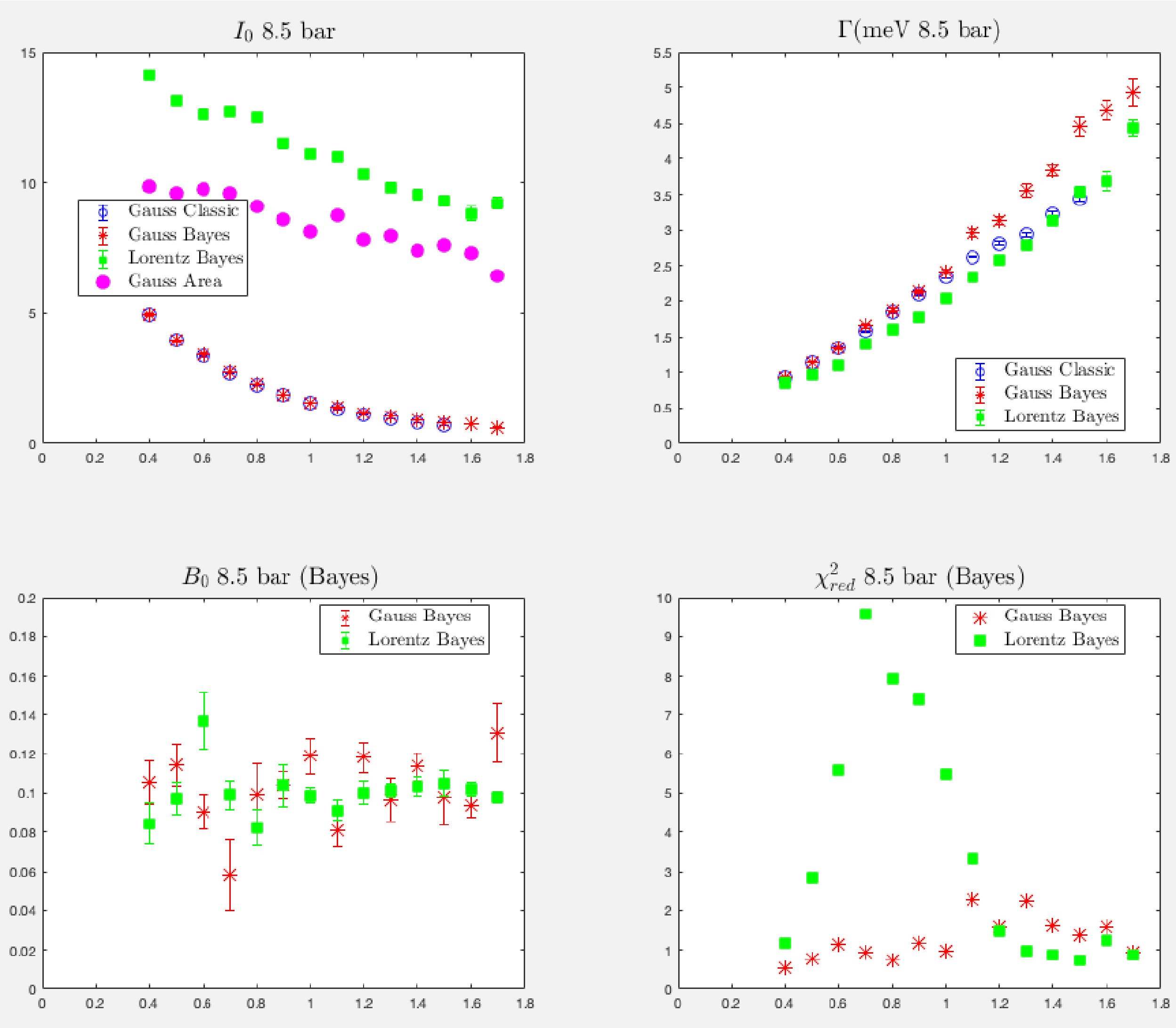

Supplementary Figure 6: Bayesian analysis results for 8.5 bar. Intensity (arb. units), half width (meV), flat background (arb. units), and reduced chi-square for the Gaussian and Lorentzian fits of the Bayesian analysis (from left to right, top to bottom), plotted as a function of Q in Å^{-1}. The parameters I_0 and Γ obtained without Bayesian analysis (those reported in the main text) are indicated as “Classic”. “Gauss Area” is given by $\sqrt{\pi/\ln(2)}I_0^{\text{Gauss}}\Gamma_G$.

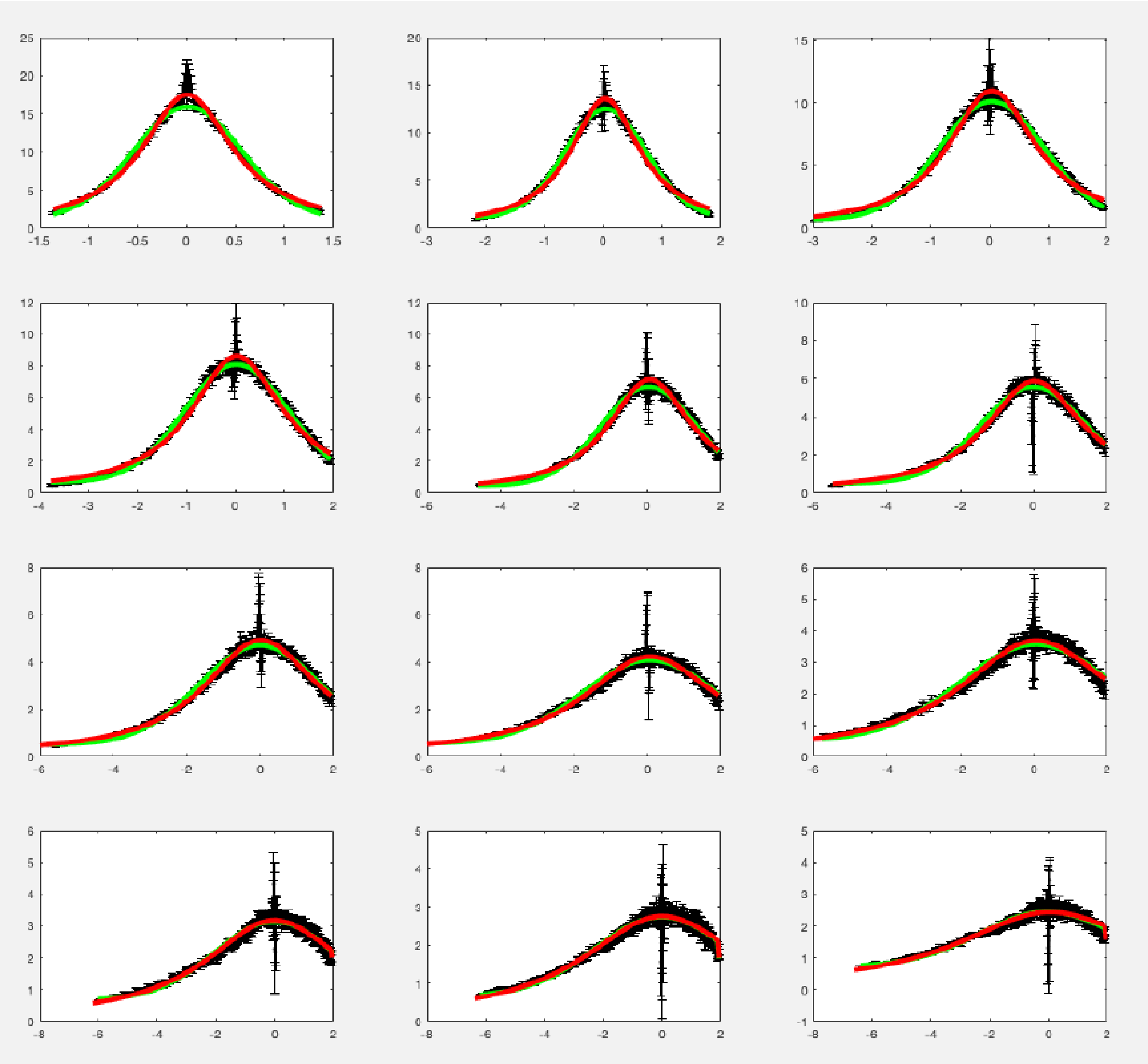

Supplementary Figure 7: Bayesian fits to the spectra at 46 bar. Gaussian (green) and Lorentzian (red) fits to the experimental spectra at 0.4 to 1.5 Å^{-1} (from left to right, top to bottom).

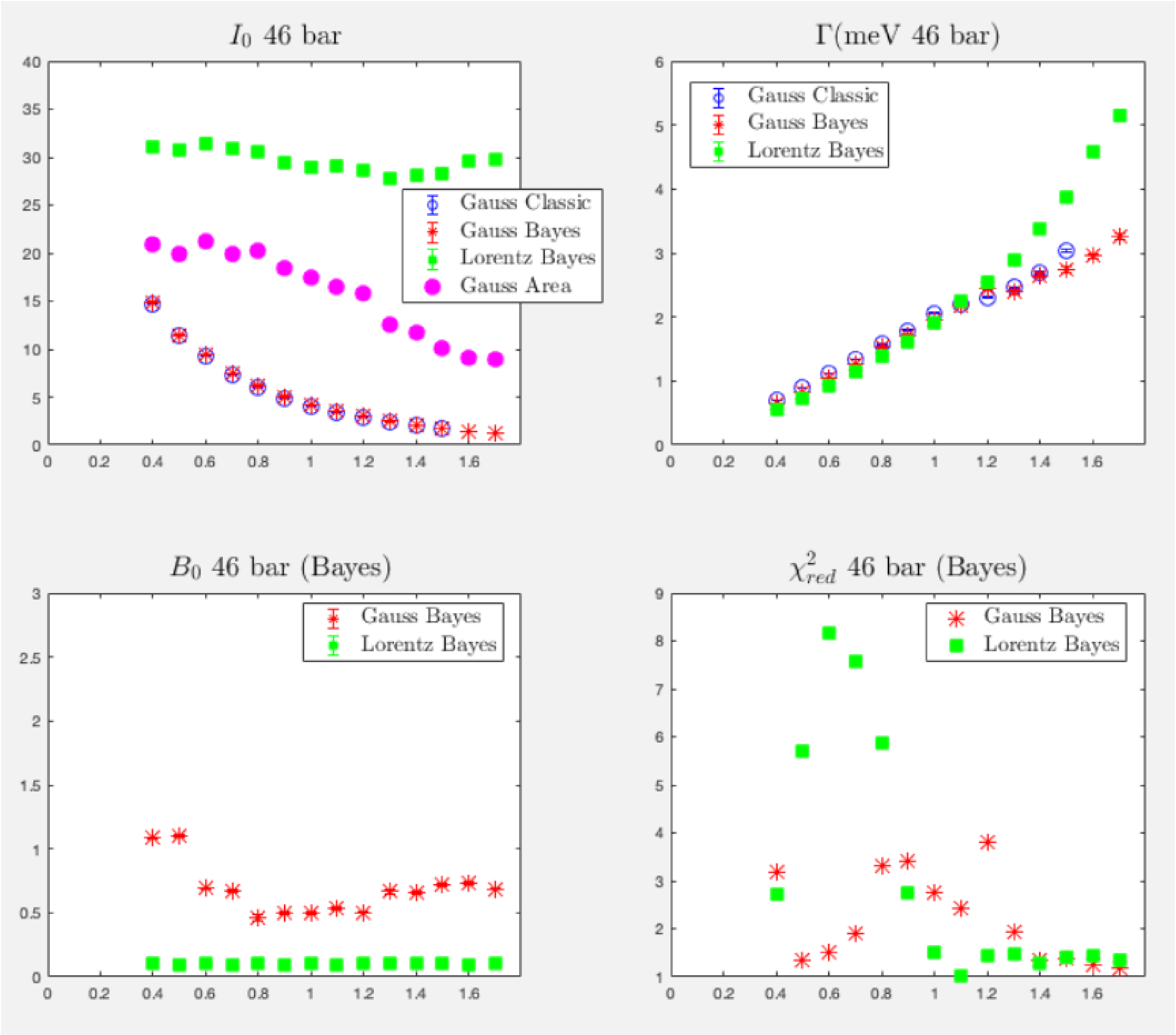

Supplementary Figure 8: Bayesian analysis results for 46 bar. Intensity (arb. units), half width (meV), flat background (arb. units), and reduced chi-square for the Gaussian and Lorentzian fits of the Bayesian analysis (from left to right, top to bottom), plotted as a function of Q in $Å^{-1}$. The parameters I_0 and Γ obtained without Bayesian analysis (those reported in the main text) are indicated as "Classic". "Gauss Area" is given by $\sqrt{\pi/\ln(2)}I_0^{\text{Gauss}}\Gamma_G$.

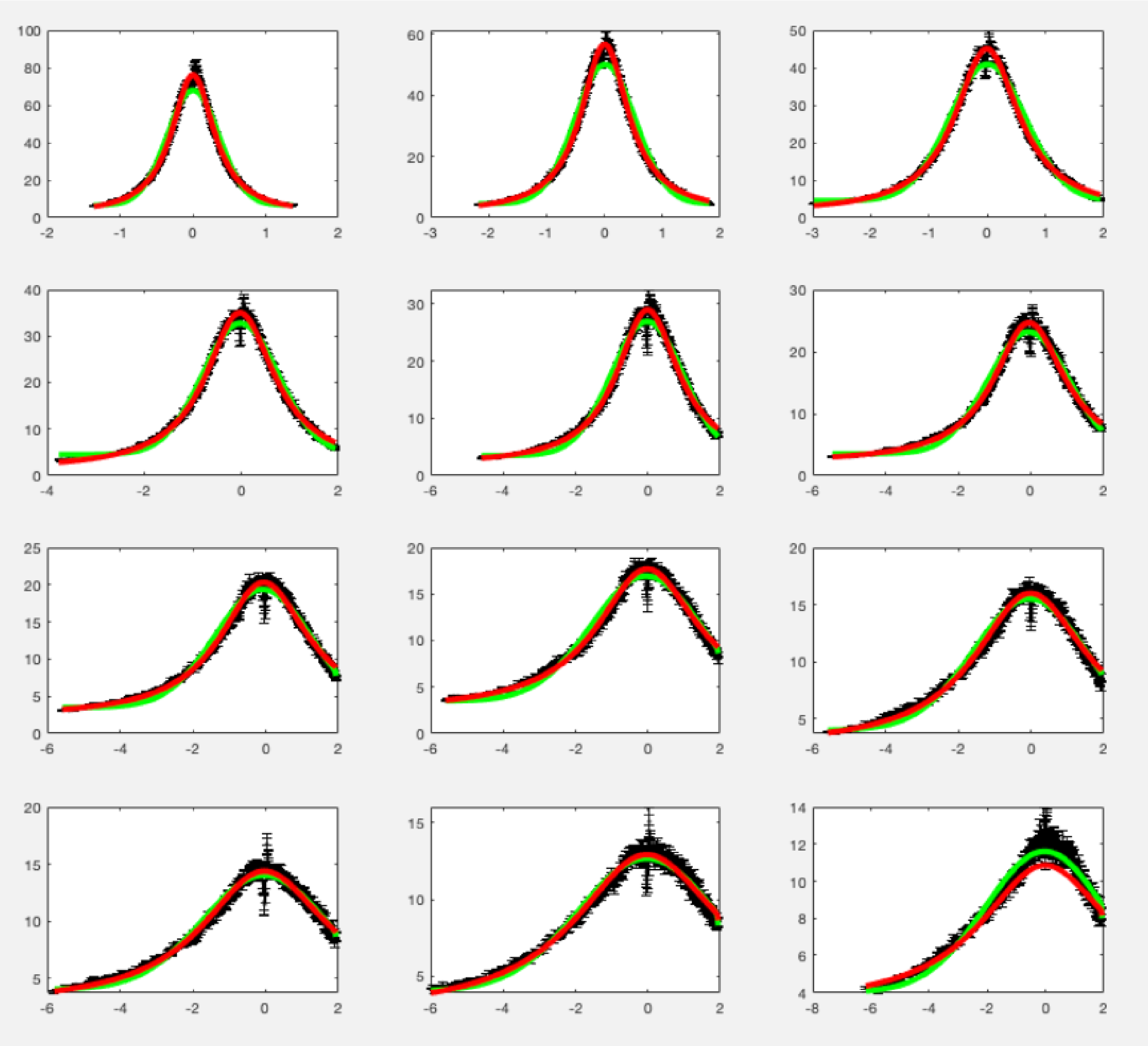

Supplementary Figure 9: Bayesian fits to the spectra at 67 bar. Gaussian (green) and Lorentzian (red) fits to the experimental spectra at 0.4 to 1.5 Å^{-1} (from left to right, top to bottom).

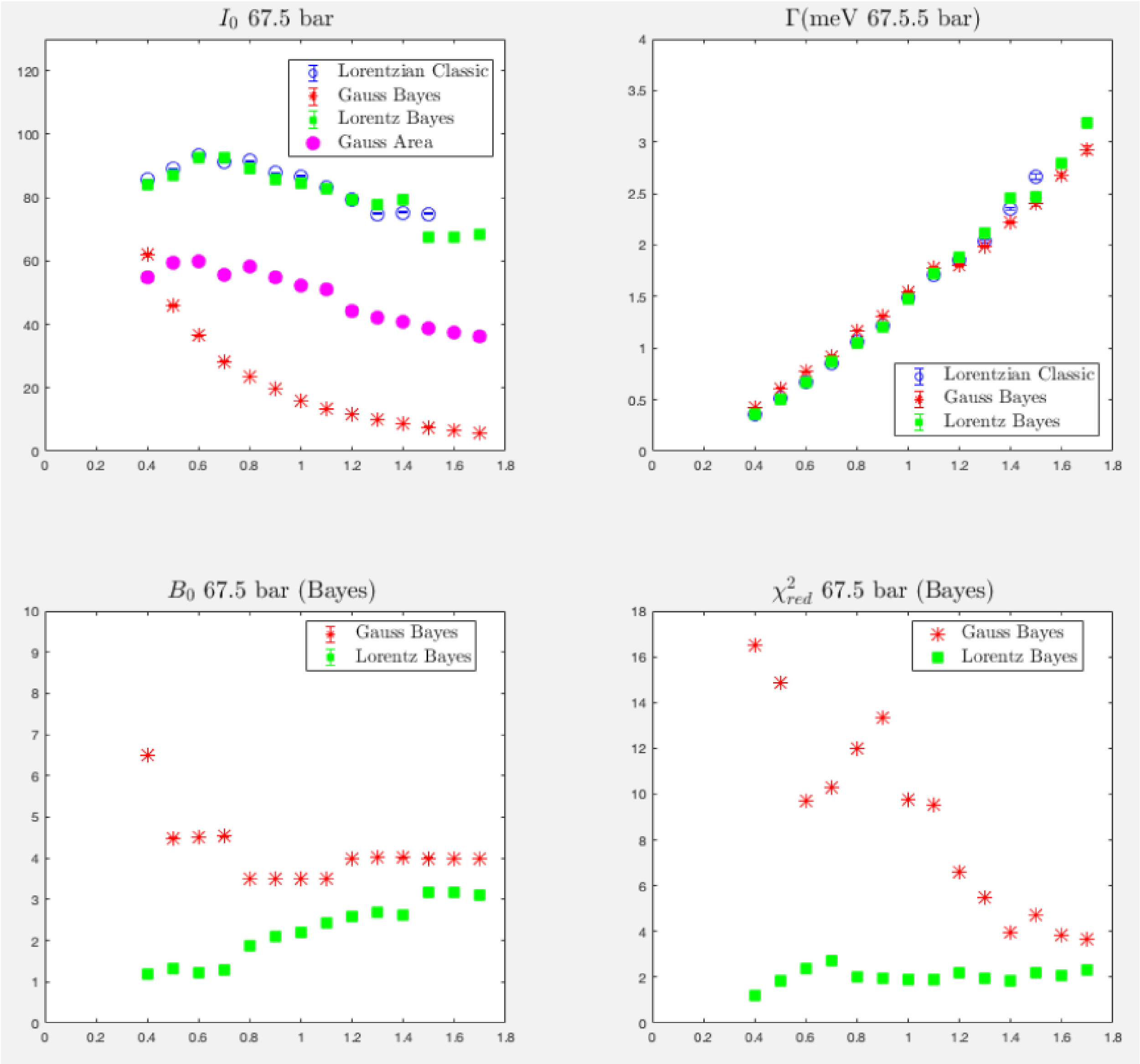

Supplementary Figure 10: Bayesian analysis results for 67 bar. Intensity (arb. units), half width (meV), flat background (arb. units), and reduced chi-square for the Gaussian and Lorentzian fits of the Bayesian analysis (from left to right, top to bottom), plotted as a function of Q in Å^{-1}. The parameters I_0 and Γ obtained without Bayesian analysis (those reported in the main text) are indicated as "Classic". "Gauss Area" is given by $\sqrt{\pi/\ln(2)}I_0^{\text{Gauss}}\Gamma_G$.

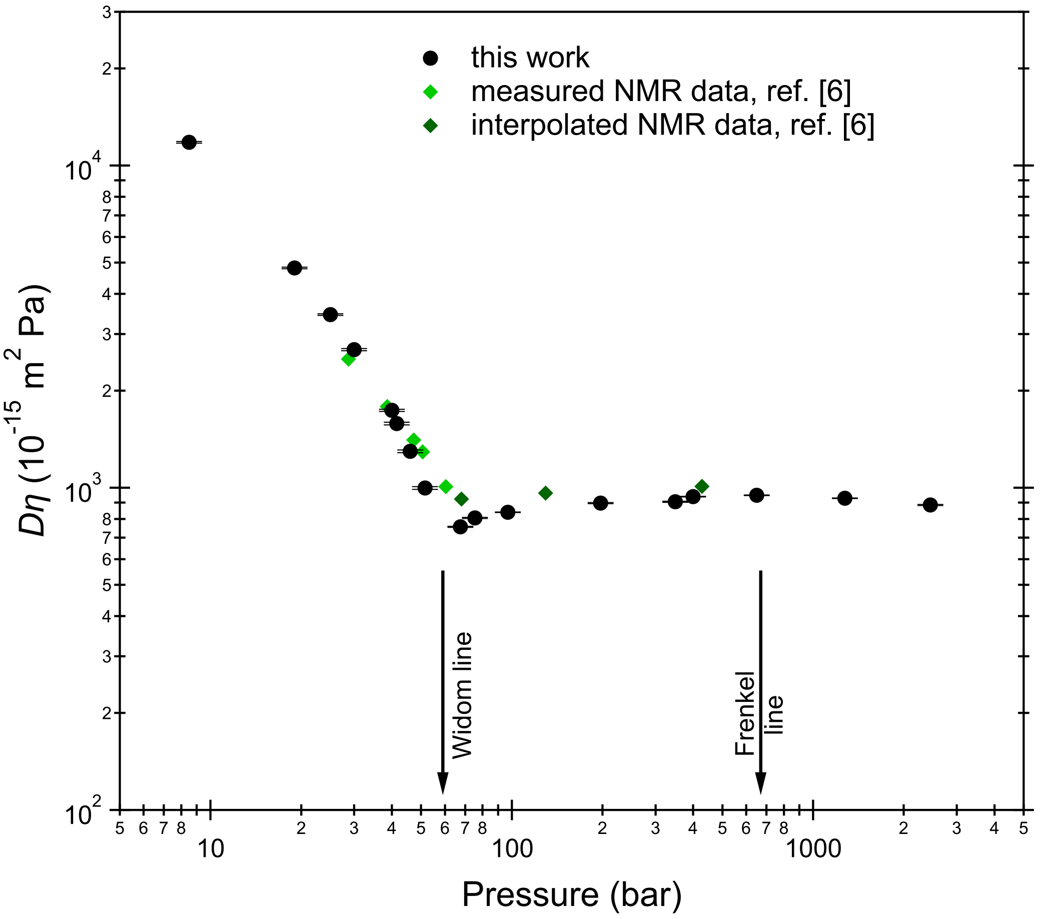

Supplementary Figure 11: Test of the Stokes–Einstein–Sutherland relation. Pressure dependence of the product between the self-diffusion coefficient D at 200 K (from this work and from ref. 6) and the viscosity η at the same temperature from the equation of state-like viscosity model of ref. 7. The error bars were obtained by propagating the errors in D only. The Stokes–Einstein–Sutherland relation predicts a constant product $D\eta$ along isotherms.

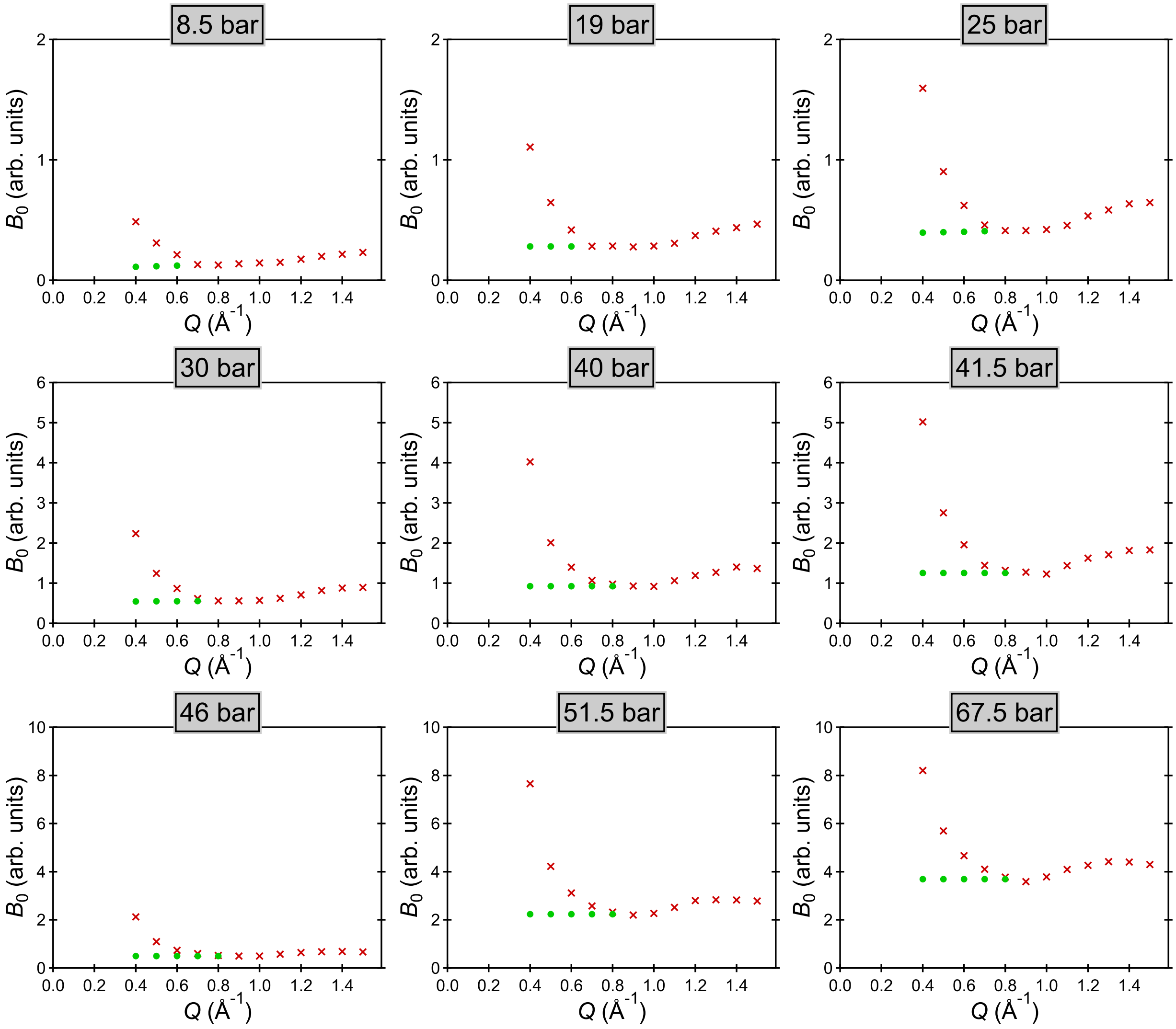

Supplementary Figure 12: Flat background parameter B_0 of the Gaussian fits. B_0 parameter of the Gaussian fits as a function of Q. Freely refined values as red crosses and imposed values as green dots.

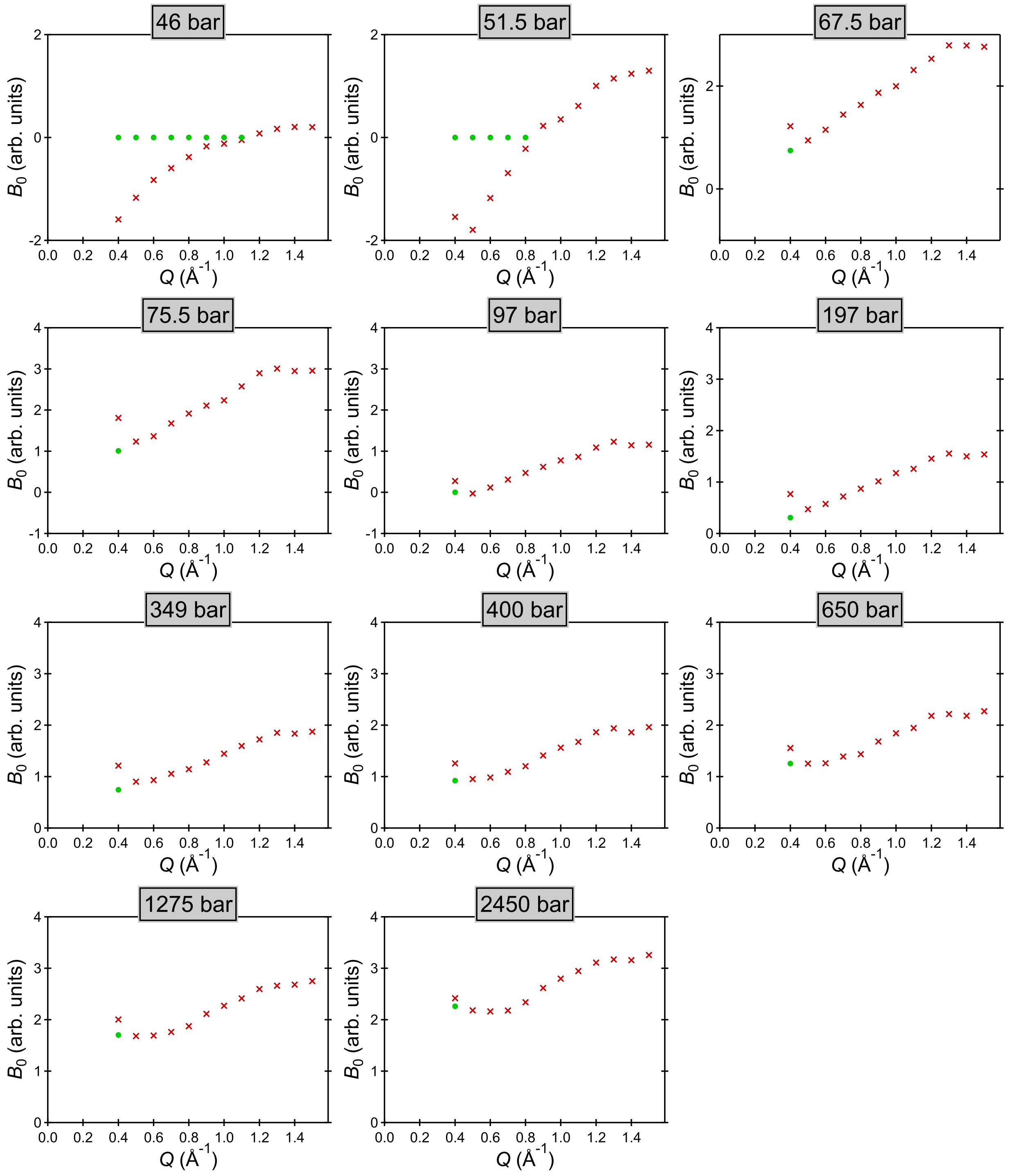

Supplementary Figure 13: Flat background parameter B_0 of the Lorentzian fits. B_0 parameter of the Lorentzian fits as a function of Q. Freely refined values as red crosses and imposed values as green dots.

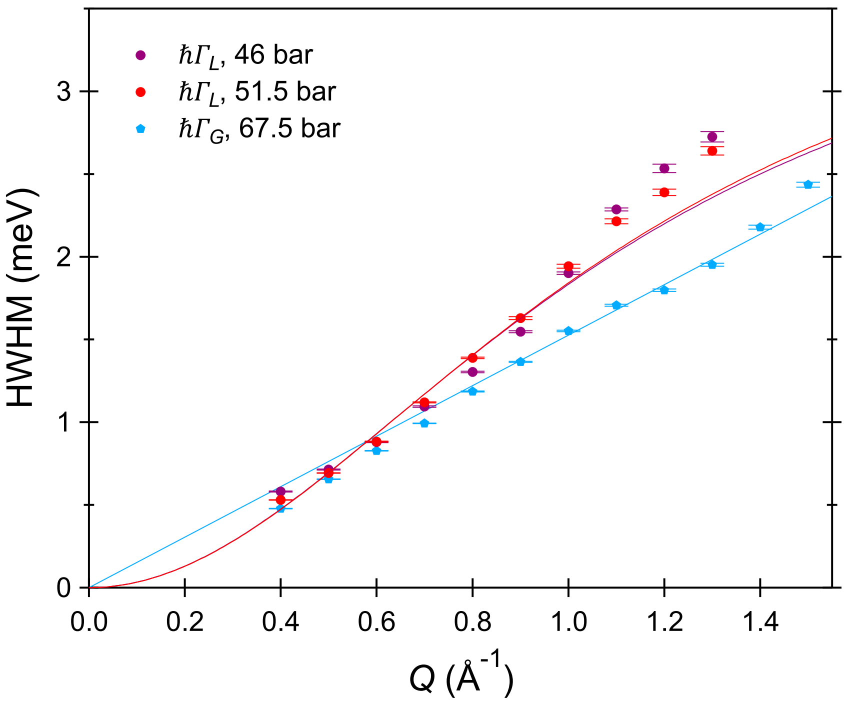

Supplementary Figure 14: Wavevector transfer dependence of Γ_G and Γ_L at other pressures. Fitted Lorentzian half widths at half maximum as a function of Q at 46 and 51.5 bar, namely pressures for which Gaussian fits were preferred, as well as fitted Gaussian half widths at half maximum as a function of Q at 67.5 bar, namely a pressure for which Lorentzian fits were preferred. The best fits to the shown data at each pressure are reported as solid lines.

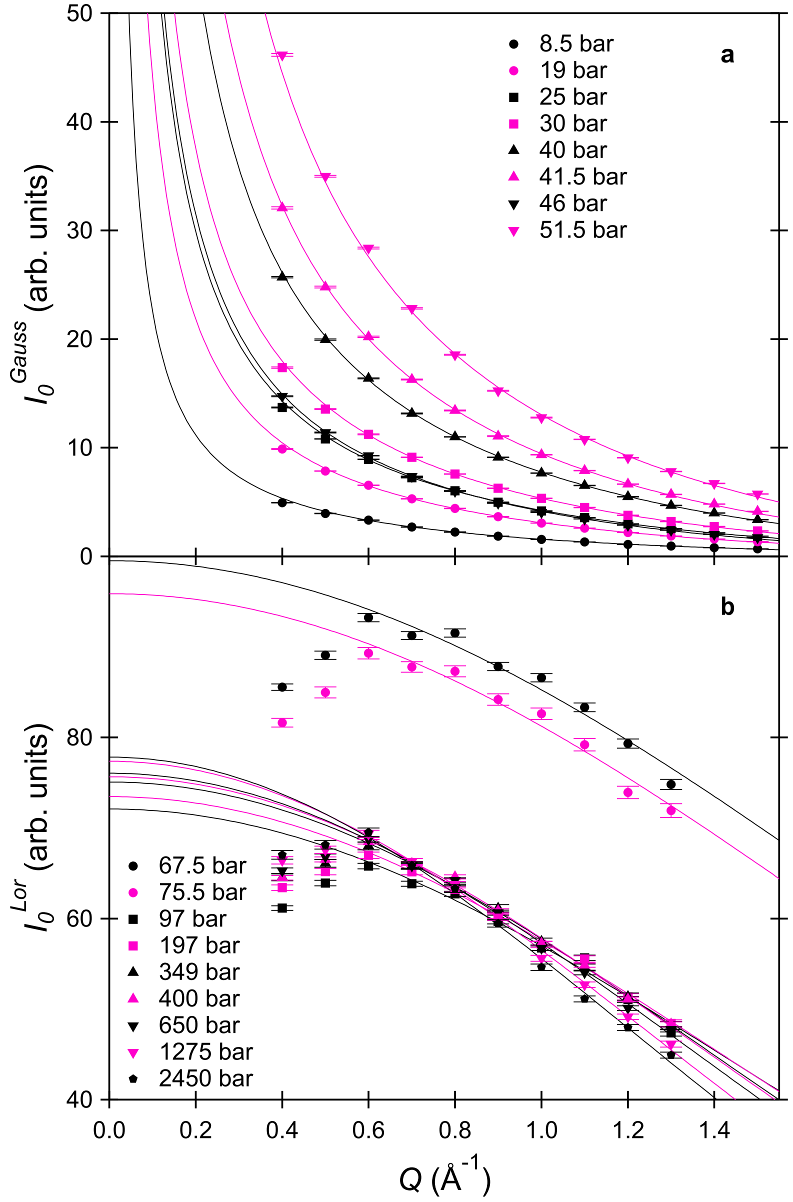

Supplementary Figure 15: Wavevector transfer dependence of I_0^{Gauss} and I_0^{Lor}. Fitted Gaussian (panel **a**) and Lorentzian (panel **b**) intensities as a function of Q at all investigated pressures. The Gaussian intensity is lower at 46 bar compared to other comparable pressures because the 46 bar data were taken with the aluminium spacer in the high-pressure cell. The Lorentzian intensity is higher at 67.5 and 75.5 bar compared to the other pressures because those data were taken without aluminium spacer. The best fits to the data at each pressure (Eqs. 9 and 10 of the main text) are shown as solid lines and provide the mean displacement $\left\langle u^2 \right\rangle^{1/2}$ of the Debye-Waller factor, which is reported in Supplementary Fig. 16. Given the large systematic deviation shown by I_0^{Lor}, the two lowest Q points were excluded in all the fits.

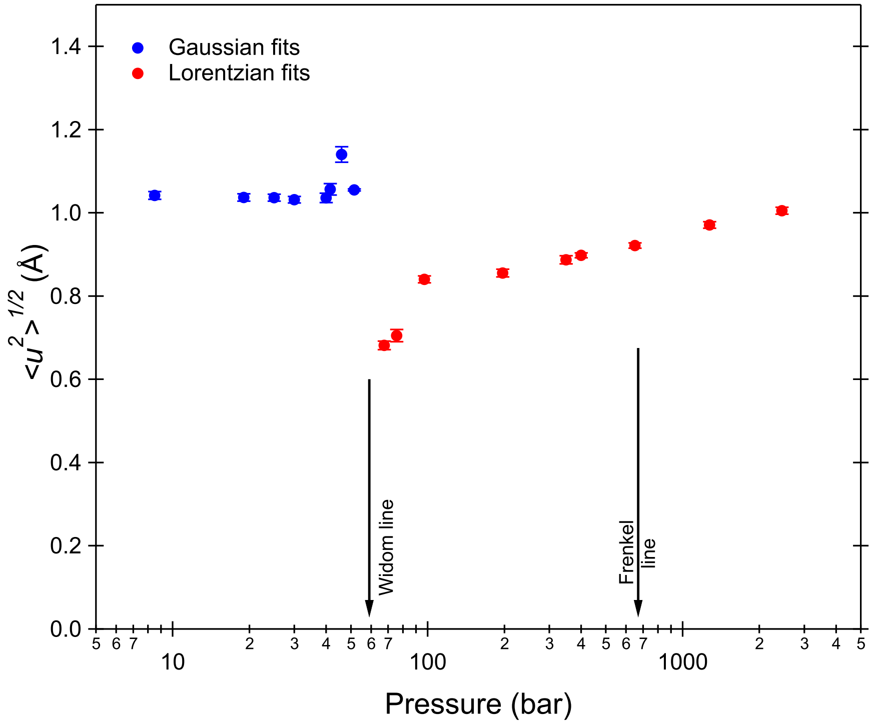

Supplementary Figure 16: Mean displacement of the Debye-Waller factor. $\langle u^2 \rangle^{1/2}$ as a function of pressure, as obtained from the fits shown in Supplementary Fig. 15.

Supplementary References

1. Pieprzyk, S., Bannerman, M. N., Brańka, A. C., Chudak, M. & Heyes, D. M. Thermodynamic and dynamical properties of the hard sphere system revisited by molecular dynamics simulation. *Phys. Chem. Chem. Phys.* **21**, 6886–6899 (2019).

2. De Francesco, A., Cunsolo, A. & Scaccia, L. Bayesian approach for x-ray and neutron scattering spectroscopy. In Cunsolo, A., Franco, M. K. K. D. & Yokaichiya, F. (eds.) *Inelastic X-Ray Scattering and X-Ray Powder Diffraction Applications*, chap. 2, 26 (IntechOpen, 2020).

3. Tierney, L. Markov chains for exploring posterior distributions. *Ann. Stat.* **22**, 1701–1762 (1994).

4. Green, P. J. Reversible jump Markov chain Monte Carlo computation and Bayesian model determination. *Biometrika* **82**, 711–732 (1995).

5. De Francesco, A., Guarini, E., Bafile, U., Formisano, F. & Scaccia, L. Bayesian approach to the analysis of neutron Brillouin scattering data on liquid metals. *Phys. Rev. E* **94**, 023305 (2016).

6. Oosting, P. H. & Trappeniers, N. J. Proton-spin–lattice relaxation and self-diffusion in methanes: IV. Self-diffusion in methane. *Physica* **51**, 418–431 (1971).

7. Younglove, B. A. & Ely, J. F. Thermophysical properties of fluids. II. Methane, ethane, propane, isobutane, and normal butane. *J. Phys. Chem. Ref. Data* **16**, 577 (1987).
